# Antibiotic-Induced Changes in Pigment Accumulation, Photosystem II, and Membrane Permeability in a Model Cyanobacterium

**DOI:** 10.3389/fmicb.2022.930357

**Published:** 2022-06-22

**Authors:** Yavuz S. Yalcin, Busra N. Aydin, Mst Sayadujjhara, Viji Sitther

**Affiliations:** Department of Biology, Morgan State University, Baltimore, MD, United States

**Keywords:** cyanobacteria, fluorescence yield, *Fremyella diplosiphon*, hormetic effect, lactate dehydrogenase, oxidative stress, β-lactams

## Abstract

*Fremyella diplosiphon* is a well-studied a model cyanobacterium for photosynthesis due to its efficient light absorption potential and pigment accumulation. In the present study, the impact of ampicillin, tetracycline, kanamycin, and cefotaxime on pigment fluorescence and photosynthetic capacity in *Fremyella diplosiphon* strains B481-WT and B481-SD was investigated. Our results indicated that both strains exposed to kanamycin from 0.2 to 3.2 mg/L and tetracycline from 0.8 to 12.8 mg/L enhanced growth and pigment accumulation. Additionally, B481-SD treated with 0.2–51.2 mg/L ampicillin resulted in a significant enhancement of pigment fluorescence. A detrimental effect on growth and pigmentation in both the strains exposed to 6.4–102.5 mg/L kanamycin and 0.8–102.5 mg/L cefotaxime was observed. Detection of reactive oxygen species revealed highest levels of oxidative stress at 51.2 and 102.5 mg/L kanamycin for B481-*SD* and 102.5 mg/L for B481-WT. Membrane permeability detected by lactate dehydrogenase assay indicated maximal activity at 0.8 mg/L ampicillin, kanamycin, and tetracycline treatments on day 6. Abundant vacuolation, pyrophosphate, and cyanophycin granule formation were observed in treated cells as a response to antibiotic stress. These findings on the hormetic effect of antibiotics on *F. diplosiphon* indicate that optimal antibiotic concentrations induce cellular growth while high concentrations severely impact cellular functionality. Future studies will be aimed to enhance cellular lipid productivity at optimal antibiotic concentrations to disintegrate the cell wall, thus paving the way for clean bioenergy applications.

## Introduction

In recent years, increased antibiotic contamination in surface and groundwater has drawn worldwide attention due to their potential consequences for the environmental ecosystem and health. Globally, antibiotic consumption has increased by 64% and at the rate of 39% over the past two decades (WHO, 2016; Browne et al., 2021). In the United States alone, about 10,000 tons per annum of antibiotics are consumed and account for ∼70% of the nation’s annual antimicrobial consumption (U.S. Food and Drug Administration, 2019). Antibiotic residues excreted in urine and feces after metabolism are directly introduced to the aquatic environments by poorly managed livestock that have direct access to surface water or indirectly by animal manure (Kümmerer, 2009; Bailey et al., 2015; Pan et al., 2021). While non-targeted antibiotic exposure on eukaryotes is minimal compared to prokaryotes, cyanobacteria are ten times more sensitive than algae to the harmful effects of antibiotics because of fragile cell structures (Norvill et al., 2016; Kulkarni et al., 2017). About 20 different kinds of antibiotics have been detected in the range of 1.26–127.49 ng/L in various aquatic environments (Kim et al., 2018). Of these, the β-lactam group, primarily the penicillin and cephalosporins, constitutes 50–60% of the most consumed antibiotics (Kapoor et al., 2017). Significant amount of β-lactams are directly excreted without any structural changes after metabolism (Snow et al., 2009). Besides, antibiotic residues are detrimental to microbial communities in aquatic ecosystems and are known to greatly impact cellular metabolism in cyanobacteria (González-Pleiter et al., 2013; Cui et al., 2020). The hormesis phenomenon (biphasic effect) in response to harmful environmental agents by low-dose stimulation and high-dose inhibition has been extensively studied (Kouda and Iki, 2010). Exposure of *Microcystis aeruginosa*to low dosages (< 20 mg/L) of erythromycin has been reported to trigger photosynthetic activity *(Fv/Fm)* (Wu et al., 2020).

Of the various cyanobacterial strains, *Fremyella diplosiphon* is a widely studied model organism known for its adaptive growth capability in varying light intensities. Besides, its ability to produce lipids and desirable essential fatty acids make it an ideal third generation biofuel agent. To our knowledge, the impact of antibiotics on *F. diplosiphon* growth and cell membrane permeability remains unknown. In this study, the impact of ampicillin, tetracycline, kanamycin, and cefotaxime on pigment accumulation, photosystem II (PSII) activity, reactive oxygen species (ROS) formation, and cell membrane permeability in *F. diplosiphon* strains B481-SD and B481-WT was investigated. Morphological alterations in cells exposed to antibiotics were observed by microscopic examinations.

## Materials and Methods

### Cyanobacterial Strains and Growth Conditions

*F. diplosiphon* strains, B481-WT obtained from the UTEX algae repository (Austin, TX, United States), and B481-SD (overexpressed strain with the sterol desaturase gene; accession MH329183) were used in this study. Cultures were grown in liquid BG-11/HEPES medium under wide-spectrum red light (650 nm) with continuous shaking at 170 rpm at 28°C in an Innova 44R shaker (Eppendorf, Hamburg, Germany) for 6 days. Light fluence rate was adjusted to 30 μmol m^–2^ s ^–1^ using the model LI-190SA quantum sensor (Li-Cor, United States). These conditions were kept constant during the study.

### Antibiotic Treatment

Three classes of antibiotics: β-lactams (ampicillin, cefotaxime), aminoglycosides (kanamycin), and tetracycline, were tested in this study. Antibiotic stock solutions (25x–100x) were prepared according to the manufacturer’s instructions and stored at -20°C. Working solutions in the range of 0.2–102.5 mg/L for ampicillin, kanamycin, cefotaxime and 410 mg/L for tetracycline were used in this study (Dias et al., 2015; Shang et al., 2015). Ampicillin, cefotaxime, and kanamycin working solutions were prepared immediately before use and diluted in ddH_2_O to the desired concentrations (Baselga-Cervera et al., 2019). Each antibiotic concentration was mixed with 5 ml *F. diplosiphon* cells adjusted to OD750 nm. Assays were performed in 96-well clear polystyrene microplate (Corning^®^Inc., NY) and cultures grown under conditions mentioned above. Three replicate treatments were maintained and the experiment was repeated twice. In order to minimize the effects of light scattering, every other well was left blank. Plates were sealed with a Breathe-Easy sealing membrane (Sigma-Aldrich, MO, Lot#MKCP8263) to prevent evaporative water loss and decrease the risk of contamination.

### Pigment and Photosynthesis Analysis in Antibiotic-Treated *Fremyella diplosiphon*

Phycocyanin and chlorophyll *a* fluorescence in antibiotic-treated and control *F. diplosiphon* were recorded every other day using a microplate reader (BioTek Synergy H1 Microplate Reader, Agilent, United States). While chlorophyll *a* fluorescence was recorded at an excitation of 420 nm and emission of 680 nm, phycocyanin was measured at an excitation of 590 nm and emissions of 650 nm (Rohaìček and Bartaìk, 1999). Fluorescence Epi-RGB mode was used for macro evaluation in 96 well plates on day 6 (Amersham Imager 680, GE Healthcare Bio-Sciences AB, Uppsala, Sweden). Minimal and maximal fluorescence yield (*Fo* and *Fm*) was measured using MINI-PAM (Walz, Effeltrich, Germany) every 48 h for 6 days after incubation in dark for 15 min. Based on these parameters PSII quantum yield (*Fv/Fo*) was calculated using the equation *Fv*/*Fo* = (*Fm*−*Fo*)/*Fo* (Wan et al., 2015).

### Reactive Oxygen Species Assay

Oxidative stress in antibiotic-treated *F. diplosiphon* strains was detected using 2’,7’-dichlorofluorescein diacetate, also known as H_2_DCFDA (EMD Chemicals, United States) (Ajiboye et al., 2017). After growth of cells in varying antibiotic concentrations for 6 days under conditions mentioned above, a fresh 20 mM DCFDA stock was prepared, and 50 μL added to 150 μl cells in a 96 well plate (Busch and Montgomery, 2015). Fluorescence intensity was measured at an excitation of 529 nm and emission of 495 nm using a microplate reader after incubation in the dark for 45 min at room temperature (BioTek Synergy H1 Microplate Reader, Agilent, United States). Three replicate treatments were maintained and the experiment repeated once.

### Lactate Dehydrogenase Assay and Microscopic Observations

The toxicity of ampicillin, tetracycline, and kanamycin on *F. diplosiphon* was assessed using the Pierce™ (LDH Cytotoxicity Assay Kit, Thermo Fisher Scientific, United States) according to the manufacturer’s protocol. Since cefotaxime resulted in cell death on day 6, it was not included for this assay. Strains B481-WT and B481-SD were grown in liquid BG-11/HEPES medium containing 0.2–102.5 mg/L ampicillin, tetracycline, and kanamycin in 10 ml vented culture flasks. Cells at an optical density of 0.2 at OD750 nm were grown under continuous shaking at 70 rpm and 30 μmol m^–2^ s^–1^ at 28°C in an Innova 44R shaker. Cells grown in the absence of antibiotics served as control. The flasks were placed in an incubator at 37°C in the dark for 24 h prior to LDH measurement. On day 6, 50 μl cultures were transferred to a 96 well plate, and 50 μL of the reaction mixture (LDH Cytotoxicity Assay Kit, Thermo Fisher Scientific, United States) was added. After incubation for 30 min. at room temperature in the dark, 50 μL of stop solution was added and mixed gently (Wejnerowski et al., 2018). Absorbance was measured at 490 nm using a microplate reader (BioTek Synergy H1 Microplate Reader, Agilent, United States) after 2 h incubation in the dark at room temperature. Three replicates per treatment were maintained and the experiment was repeated. On day 6, microscopic observations were made using a cytation 5 Cell Imaging Multi-Mode reader (BioTek^®^ Instruments, Inc., Winooski, United States).

### Statistical Analysis

Repeated ANOVA and Tukey’s multiple comparison tests including Pearson’s correlation were used to analyze *F. diplosiphon* sensitivity to different antibiotic treatments at each sampling point. SPSS 28.0 (IBM Corporation, Armonk, United States) was also used to analyze and plot the data.

## Results

### Antibiotics Impact Pigment Fluorescence in *Fremyella diplosiphon* Strains

**FIGURE 1:**
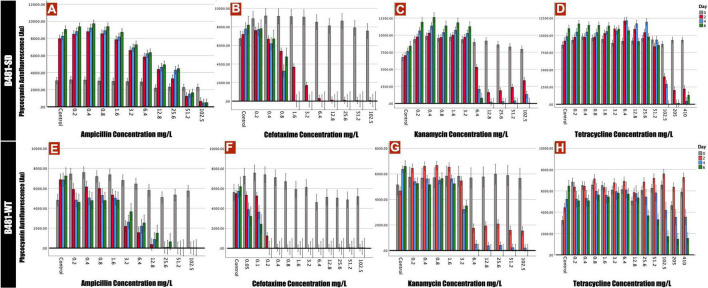
Phycocyanin autofluorescense of B481-SD and B481-WT strains reflected by 590 nm excitation and 650 nm emission. B481-SD strain was exposed to concentrations ranging from 0.2 to 102.5 mg/L ampicillin, cefotaxime, kanamycin, and tetracycline **(A–D)**, and B481-WT strain to ampicillin and kanamycin concentrations ranging from 0.2 to 102.5 mg/L; 0.05–102.5 mg/L for cefotaxime and 0.2–410 mg/L for tetracycline **(E–H)**. Both strains were cultivated in antibiotics for 6 days at 28°C with a light intensity of 30 μmol m^– 2^ s^–1^. Mean and standard deviations are indicated by error bars.

**FIGURE 2:**
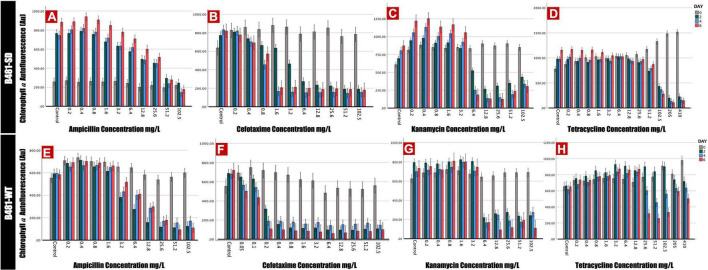
Chlorophyll *a* autofluorescence of *Fremyella diplosiphon* strains B481-SD and B481-WT reflected by excitation of 420 nm and emission of 680 nm. B481-SD strain was exposed to ampicillin, cefotaxime, kanamycin, and tetracycline concentrations ranging from 0.2 to 102.5 mg/L **(A–D)**, B481-WT strain was exposed to ampicillin and kanamycin concentrations ranging from 0.2 to 102.5 mg/L; 0.05–102.5 mg/L for cefotaxime and 0.2–410 mg/L for tetracycline **(E–H)**. Both strains are cultivated with four antibiotics for 6 days at 28°C with a light intensity of 30 μmol m^–2^ s^–1^. Mean and standard deviations are indicated by error bars.

Phycocyanin and chlorophyll *a* pigment autofluorescence was quantified to evaluate the effect of antibiotics on *F. diplosiphon* growth. Strain B481-SD treated with ampicillin ranging from 0.2 to 25.6 mg/L exhibited significant increases in phycocyanin and chlorophyll *a* autofluorescence; however, a significant decrease was detected at 51.2 and 102.5 mg/L (*p* < 0.01) (Figures 1A, 2A). By contrast, B481-WT treated with ampicillin exhibited a significant decrease in pigment autofluorescence from 3.2 to 102.5 mg/L compared to the untreated control. We observed a significant reduction of pigment autofluorescence in B481-SD and B481-WT treated with cefotaxime ranging from 0.8 to 102.5 mg/L and 0.2 to 102.5 mg/L respectively (Figures 1B,F, 2B,F). A significant reduction in phycocyanin and chlorophyll *a* autofluorescence was observed in both strains exposed to kanamycin from 6.4 to 102.5 mg/L when compared to the untreated control. However, a significant increase in B481-SD autofluorescence at lower kanamycin concentrations of 0.2–3.2 mg/L when compared to the control was observed (Figures 1C, 2C). B481-SD treated with tetracycline reduced phycocyanin and chlorophyll *a* autofluorescence at concentrations ranging from 102.5 to 410 mg/L, while it ranged from 25.6 to 410 mg/L for B481-WT. A significant increase in pigment autofluorescence was observed in both strains treated with tetracycline from 0.8 to 12.8 mg/L on day 6 (Figures 1D,H, 2D,H).

### Photosynthetic Efficacy of *Fremyella diplosiphon* Exposed to Varying Antibiotic Concentrations

**FIGURE 3:**
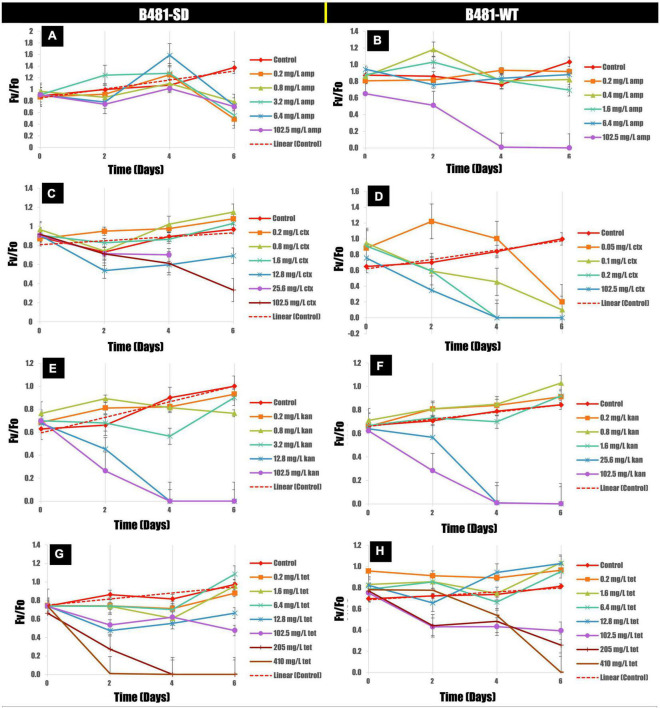
Variance of *Fv/Fo* in B481-SD **(A,C,E,G)** and B481-WT **(B,D,F,H)** strains of *Fremyella diplosiphon* in response to ampicillin, cefotaxime, kanamycin, and tetracycline exposure for 6 days. Mean and standard deviations are indicated by error bars.

Quantification of photosynthetic efficiency (*Fv/Fo*) revealed a significant increase in B481-SD strain treated with ampicillin at 0.2–3.2 mg/L on day 4 compared to the control (Figure 3A). On the other hand, a significant reduction of *Fv/Fo* ratios was observed in B-481-SD treated with 51.2 and 102.8 mg/L ampicillin on day 6 (Figures 3A,B). A substantial reduction in the *Fv/Fo* ratios in cefotaxime-treated cells was observed, with no recovery of B481-SD and B481-WT at concentrations higher than 1.6 mg/L and 0.05 mg/L respectively (Figures 3C,D). While we observed a significant decrease in *Fv/Fo* ratio in both strains treated with kanamycin from 1.6 to 102.5 mg/L, a significant increase in B481-WT at concentrations ranging from 0.2 to 1.6 mg/L kanamycin compared with the control group was noted (Figure 3F). We also observed a decrease in *Fv/Fo* ratios in both strains exposed to tetracycline concentrations higher than 102.5 mg/L (*p* < 0.05) (Figures 3G,H).

### Detection of Reactive Oxygen Species in Antibiotic-Treated *Fremyella diplosiphon*

**FIGURE 4:**
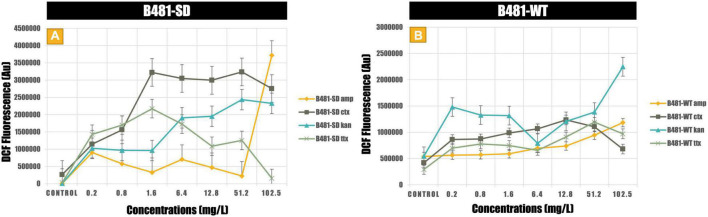
Reactive oxygen species generated in B481-SD **(A)** and B481-WT **(B)** *Fremyella diplosiphon* strains exposed to ampicillin, cefotaxime, kanamycin, and tetracycline. Mean and standard deviations are indicated by error bars.

Antibiotic-induced ROS measured using the dichlorodihydrofluorescein revealed significantly higher levels (*p* < 0.01) in both strains treated with cefotaxime, kanamycin, and tetracycline from 0.2 to 102.5 mg/L compared to the untreated control (Figures 4A,B). While B481-SD treated with 102.5 mg/L ampicillin exhibited significantly higher ROS (*p* < 0.01), it ranged from 6.4 to 102.5 mg/L for B481-WT (Figures 4A,B). Highest levels of oxidative stress were observed at 51.2 and 102.5 mg/L kanamycin for B481-SD and 102.5 mg/L for B-481-WT.

### Membrane Integrity in Antibiotic-Treated *Fremyella diplosiphon*

**FIGURE 5:**
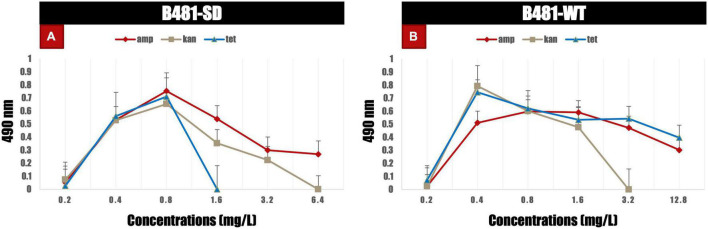
Lactate dehydrogenase activity in B481-SD and B481-WT strains of *Fremyella diplosiphon* exposed to varying concentrations of ampicillin, kanamycin, and tetracycline **(A,B)** on day 6. Mean and standard deviations are indicated by error bars.

**FIGURE 6:**
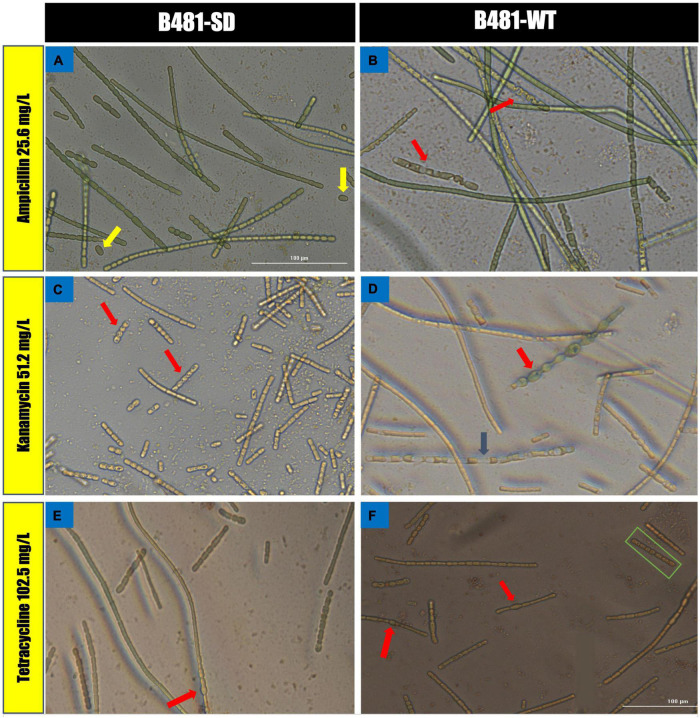
Morphological alterations of *Fremyella diplosiphon* strains in concentrations at 25.6 mg/L ampicillin **(A,B)**, 51.2 mg/L kanamycin **(C,D)** and 102.5 mg/L tetracycline **(E,F)** treatments. Representative sections of color bright field images were captured using a cytation 5 Cell Imaging Multi-Mode reader at 40× magnification bars, 100 μm.

B481-SD and B481-WT strains treated with ampicillin, tetracycline, and kanamycin exhibited maximum LDH activity at the concentrations of 0.8 and 0.4 mg/L. Specifically, enhanced LDH activity (*p* < 0.05) was observed in B481-SD treated with tetracycline, ampicillin, and kanamycin from 0.2 to 0.8 mg/L (Figure 5A). The LDH activity of B481-WT was higher in kanamycin and tetracycline at 0.4 mg/L compared to ampicillin at the same concentration (Figure 5B). Microscopic observations such as filament fragmentation and alteration of cell shape were observed at concentrations higher than 25.6 mg/L ampicillin for B481-WT and 51.2 mg/L kanamycin for B481-SD and B481-WT (Figures 6B–D). In addition, cellular stress-related structures such as pyrophosphate granules (Figure 6F, green rectangle), akinetes (Figure 6A, yellow arrows), and cellular vacuoles (Figures 6B–F, red arrows) were observed in the strains exposed to higher antibiotic concentrations.

## Discussion

In the present study, we evaluated the effect of four different antibiotics on pigment autofluorescence and photosynthetic activity in two *F. diplosiphon* strains. Additionally, we observed membrane permeability and intracellular ROS production to determine the effect of antibiotic treatment on the strains.

### Alterations of Pigment Autofluorescence in Antibiotic-Treated *Fremyella diplosiphon*

Of the four antibiotics tested in this study, ampicillin and cefotaxime belonged to the β-lactam group and are known to bind to the penicillin-binding protein of the prokaryotic cell (Stokes et al., 2019). The structural resemblance of the *F. diplosiphon* cell wall to gram-negative prokaryotes explains the sensitivity of cyanobacteria to β-lactam antibiotics (Springstein et al., 2020). Interestingly, phycocyanin and chlorophyll *a* autofluorescence in B481-SD was enhanced at lower ampicillin concentrations (0.2–25.6 mg/L); however, its growth was significantly inhibited at concentrations above 51.2 mg/L. On the other hand, a substantial decline in pigment fluorescence was observed in B481-WT at concentrations higher than 3.2 mg/L ampicillin. Thus, significantly higher pigment fluorescence in B481-SD at these concentrations provide further evidence of the sterol dehydrogenase gene overexpression associated with ampicillin resistance. It is also known that the molecular configuration against β-lactam antibiotics is a mechanism for acquiring bacterial resistance (Alpay-Karaoglu et al., 2007; Sanganyado and Gwenzi, 2019). The significant reduction of pigment accumulation observed in cefotaxime at concentrations above 1.6 mg/L for B481-SD and 0.2 mg/L for B481-WT indicate the higher sensitivity of B481-WT to cefotaxime. Additionally, enhanced fluorescence of the strains exposed to kanamycin (0.2–3.2 mg/L) and tetracycline (0.8–12.8 mg/L) on day 6 indicate the hormetic effect of these antibiotics on stimulation and inhibition. Our results are in accordance with the findings of Liu et al. (2015), who reported the toxic effect of amoxicillin at concentrations higher than 6.88 μg/L in *Microcystis aeruginosa*, while a growth-stimulating effect was observed at concentrations below. A similar biphasic effect was reported in *Skeletonema costatum* where exposure to florfenicol at < 2.0 mg/L enhanced growth while inhibition was reported at > 2.0 mg/L (Liu et al., 2012). As noted in our study, ampicillin, kanamycin, and tetracycline at higher concentrations inhibited *F. diplosiphon* pigment autofluorescence (Figures 1, 2). Despite lowering the cefotaxime concentrations to 0.05 mg/L (Figures 1, 2B,F), we did not observe the hormetic effect in B481-WT. Exposure to kanamycin and tetracycline resulted in approximately twice more remarkable pigment fluorescence in B481-SD than B481-WT, indicating higher tolerance of B481-SD to harmful antibiotics regardless of β-lactam resistance. In a previous study by Fathabad et al. (2019), a 27.2% enhancement of total lipid content in B481-SD compared to B481-WT was reported, which was attributed to gene overexpression. The enhanced lipid production in B481-SD could be attributed to sustained membrane integrity, due to increased fatty acids that facilitates membrane repair in B48-SD (Sen, 2020). On the other hand, kanamycin and tetracycline inhibit protein synthesis by binding to the bacterial ribosomal subunit resulting in a misreading of the t-RNA. The inhibition of RNA translation could have resulted in protein-derived *F. diplosiphon* pigments as observed in our study. In a report by Daghrir and Drogui (2013), tetracycline at 100 mg/L was reported to be detrimental to aquatic organisms. Although photocatalytic degradation of tetracycline occurs in a few days, the byproducts anhydrotetracycline and 4-epianhydrotetracycline have been reported to be more toxic than the primary compound (Halling-Sørensen et al., 2002). Thus, high concentrations of tetracycline ranging from 205 to 410mg/L were selected in this study. These residues might have also contributed to the pigment fluorescence of *F. diplosiphon*, as observed in our study.

### Antibiotic Exposure Enhance *Fremyella diplosiphon* Membrane Permeability

Assessment of membrane integrity as a measure of extracellular LDH enzyme activity revealed a linear correlation (data not shown). In both strains treated with antibiotics, LDH activity correlated to phycocyanin and chlorophyll *a* accumulation in a dose-dependent manner, indicating a positive correlation between increased metabolic activity and membrane permeability prior to complete cell destruction. Antimicrobial agents such as ampicillin and cefotaxime that target prokaryotic cell walls are reported to be more effective in weakening membranes than ribosomal inhibitor antibiotics such as kanamycin and tetracycline (Stokes et al., 2019). These compounds are known to trigger intracellular signaling *via* the shifting of metabolic compounds such as glucose, glycerol, and pyruvate to lipid synthesis. In a study by Liu et al. (2011) weakening of the cell wall by ampicillin was reported to facilitate free fatty acids (FFA) secretion by reducing feedback inhibition of enzymes involved in the synthesis of fatty acid precursors, thus resulting in an overall increase in FFA production (Alpay-Karaoglu et al., 2007). Enhanced lipid production in *Synechocystis* species was correlated to a loss in membrane components as well (Oliveira et al., 2016). Thus, we hypothesize that membrane damage could lead to permeability changes as indicated by increased metabolic activity stimulating lipid synthesis and accumulation.

Production of ROS is pertinent when antibiotic concentrations above the threshold level can onset cellular stress (Chen et al., 2019). In cellular metabolism, a dynamic equilibrium between ROS generation and elimination is maintained due to the operation of antioxidant defense systems. In addition, a tremendous increase of ROS may cause oxidative damage resulting in cell injury and ultimately cell death due to protein and lipid damage and impairment of cyanobacterial homeostasis (Du et al., 2018). The reactive oxygen radicals generated have the potential to react with membrane lipids and protein (phycocyanin, chlorophyll a) structures in cyanobacteria. Our results revealed maximal ROS levels in both strains treated with ampicillin at 102.5 mg/L, with lower ROS levels in B481-SD compared to B481-WT. While B481-SD treated with cefotaxime at 0.2–102.5 mg/L exhibited higher levels of ROS, it was not detected in B481-WT (Supplementary Figure 1D). It is possible that a reduction in the number of viable cells could have lowered ROS production in B481-WT. We hypothesize that the detrimental effect of cefotaxime could have resulted in higher ROS levels in the first few days of the experiment, while the unstable structure of oxygen radicals due to easy loss of unpaired electrons and elimination could be responsible for lower ROS at the end of the testing period. Both strains exposed to kanamycin at 102.5 mg/L revealed maximum ROS on day 6 and exhibited a similar growth paradigm at the concentrations tested. ROS production is generally caused by the leakage of electrons from the photosystem electron transport chain as part of the metabolism of photosynthetic organisms and plays a dynamic equilibrium in the operation of antioxidant defense system. A study on the analysis of the impact of superoxide dismutase (SOD) enzymes in 149 cyanobacterial strains has shown diverse SOD enzyme isoforms, indicating that the antioxidant mechanism that eliminates ROS could vary in different cyanobacterial strains (Boden et al., 2021).

Chlorophyll *a* is an important light-harvesting photosynthetic pigment in cyanobacteria, which plays a crucial role in energy absorption and transduction (Latifi et al., 2009). The electron transport capacity of photosynthetic pigments such as chlorophyll *a* is closely related to the quality of the photosynthetic apparatus and indicated by PSII activity (*Fv/Fo*). As the pigment content of cyanobacteria decrease, the thylakoid membrane becomes the active site due to cell wall damaging antibiotics such as penicillin and cephalosporins (Liu et al., 2015). The decrease in PSII activity of both strains at higher ampicillin, cefotaxime, and kanamycin concentrations of 51.2 and 102.5 mg/L on day 6 as observed in our study could be attributed to enhanced ROS production. Therefore, it is possible that reduced pigment functions could have occurred due to ROS-induced damage to the thylakoid membranes, particularly chlorophyll *a*.

## Conclusion

In this study, we investigated the effect of four antibiotics at varying concentrations on phycocyanin and chlorophyll *a* autofluorescence of *F. diplosiphon* strains. Significant increases in pigment accumulation at specific antibiotic concentrations pave the way for further studies to accomplish lipid synthesis for easy and efficient biofuel production. Future studies will aim toward enhancing membrane permeability in the B481-SD strain with antibiotics. In addition, the combined effect of antibiotics and zero-valent iron nanoparticles in enhancing specific lipid gene overexpression and transcript levels activity will be studied.

## Data Availability Statement

The original contributions presented in this study are included in the article/Supplementary Material, further inquiries can be directed to the corresponding author.

## Author Contributions

YY and BA designed and performed the experiments, analyzed, interpreted the data, and drafted the manuscript. MS gave critical comments on the article. VS designed, conceived the study, edited the manuscript, and obtained funding. All authors read and approved the final manuscript.

## Conflict of Interest

The authors declare that the research was conducted in the absence of any commercial or financial relationships that could be construed as a potential conflict of interest.

## Publisher’s Note

All claims expressed in this article are solely those of the authors and do not necessarily represent those of their affiliated organizations, or those of the publisher, the editors and the reviewers. Any product that may be evaluated in this article, or claim that may be made by its manufacturer, is not guaranteed or endorsed by the publisher.
