# Supplementary figures and images for: Antibiotic-Induced Changes in Pigment Accumulation, Photosystem II, and Membrane Permeability in a Model Cyanobacterium
Source: Front Microbiol. 2022 Jun 22;13:930357. doi: 10.3389/fmicb.2022.930357 (PMC9257187; doi:10.3389/fmicb.2022.930357)

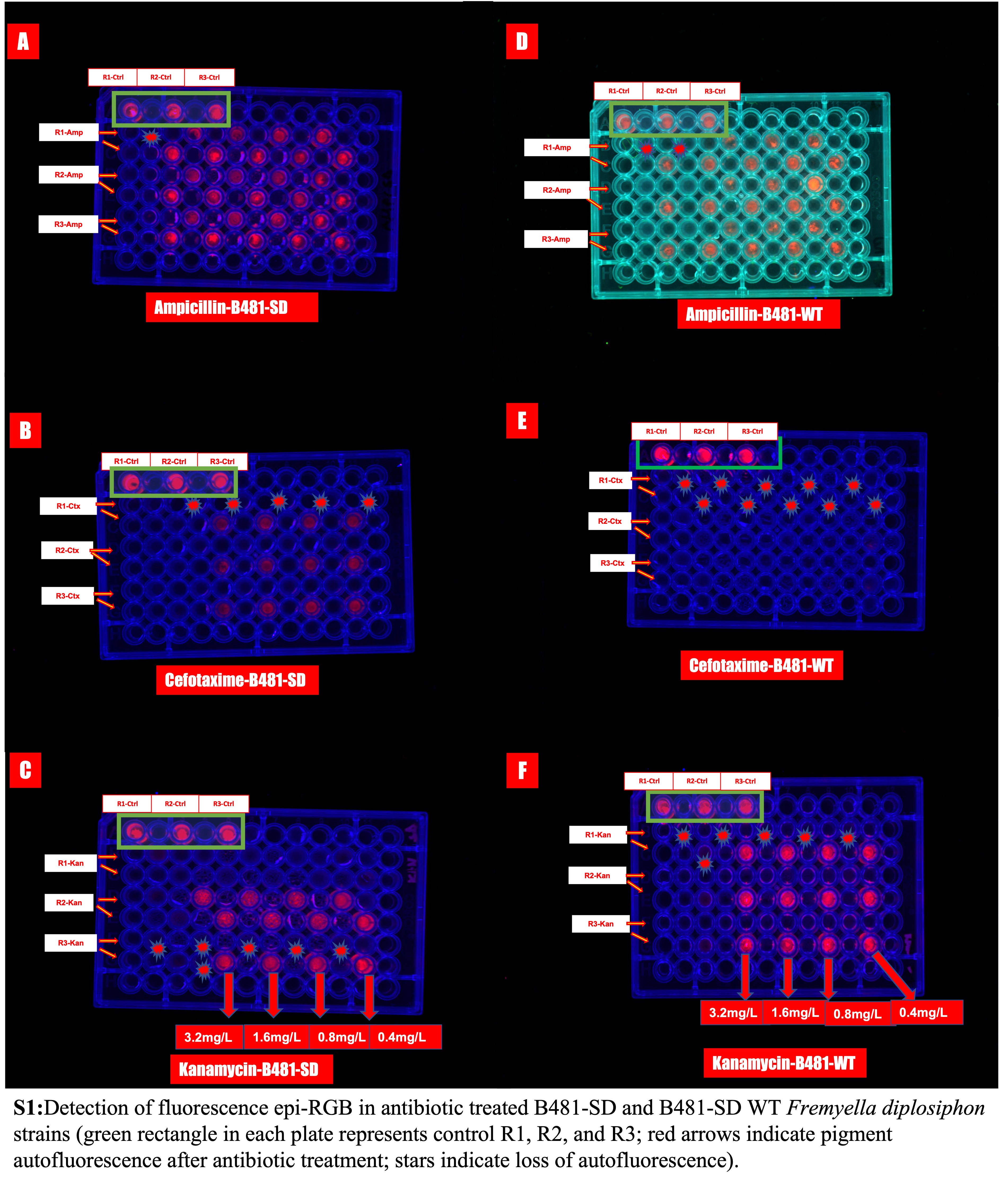

Supplement: Supplementary file 1 [file Image_1.JPEG]
